# Patients values regarding primary health care: a systematic review of qualitative and quantitative evidence

**DOI:** 10.1186/s12913-023-09394-8

**Published:** 2023-04-25

**Authors:** Agnes Bhakti Pratiwi, Retna Siwi Padmawati, Joko Mulyanto, Dick L. Willems

**Affiliations:** 1 Department of Ethics, Law, and Humanities, Faculty of Medicine, Amsterdam University Medical Center, University of Amsterdam, Meibergdreef 9, 1105AZ Amsterdam, The Netherlands; 2 Department of Medical Education and Bioethics, Faculty of Medicine, Public Health and Nursing, Universitas Gadjah Mada, Yogyakarta, Indonesia; 3 Department of Health Behavior, Environment, and Social Medicine, Faculty of Medicine, Public Health and Nursing, Universitas Gadjah Mada, Yogyakarta, Indonesia; 4 Center for Bioethics and Medical Humanities, Faculty of Medicine, Public Health and Nursing, Universitas Gadjah Mada, Yogyakarta, Indonesia; 5 Department of Public Health and Community Medicine, Faculty of Medicine, Universitas Jenderal Soedirman, Purwokerto, Indonesia; 6 Department of Public and Occupational Health, Amsterdam University Medical Center, University of Amsterdam, Amsterdam, The Netherlands

**Keywords:** Patient values, Patient preference, Patient empowerment, Primary health care, Healthcare access, Universal health coverage

## Abstract

**Background:**

Accessible and high-quality primary health care (PHC) is fundamental to countries moving towards universal health coverage. In order to improve the quality of patient-centered care provided in PHC, a comprehensive understanding of patients’ values is crucial to address any gaps in the health care system. This systematic review aimed to identify patients’ values relevant to PHC.

**Methods:**

We searched primary qualitative and quantitative studies about patients’ values related to primary care in PubMed and EMBASE (Ovid) from 2009 to 2020. The studies’ quality was assessed using Joanna Briggs Institute (JBI) Critical Appraisal Checklist for both quantitative and qualitative studies and Consolidated Criteria for Reporting Qualitative Studies (COREQ) for qualitative studies. A thematic approach was used in the data synthesis.

**Outcome:**

The database search resulted in 1,817 articles. A total of 68 articles were full-text screened. Data were extracted from nine quantitative and nine qualitative studies that met the inclusion criteria. The participants of the studies were mainly the general population in high-income countries. Four themes emerged from the analysis: patients’ values related to privacy and autonomy; values associated with the general practitioners including virtuous characteristics, knowledge and competence; values involving patient-doctor interactions such as shared decision-making and empowerment; and core values related to the primary care system such as continuity, referral, and accessibility.

**Conclusions:**

This review reveals that the doctor’s personal characteristics and their interactions with the patients are critical considerations concerning the primary care services from the patients’ point of view. The inclusion of these values is essential to improve the quality of primary care.

**Supplementary Information:**

The online version contains supplementary material available at 10.1186/s12913-023-09394-8.

## Introduction

Different countries are moving health systems resources towards universal health coverage, necessitating efficient health resources allocation, sustainable health financing, and a strong primary healthcare (PHC) system as the backbone [1–3]. The role of PHC has become indispensable because it serves as the initial and continuous contact for patients, acts as the gatekeeper to higher levels of care, and provides a coordinated and comprehensive care to the community [4]. The PHC principles are universal access, equitable care provision, accentuating prevention, health promotion, and community participation [1]. In the practice of PHC service provision, it is imperative to find the balance in social and medical aspects to meet the need of its users.

However, from the patients’ point of view, PHC may not fully satisfy their needs. Evidence suggest that even in the absence of major barriers such as costs and geography, some people would prefer other healthcare services, such as emergency departments, hospitals, or traditional healers [5–8]. A perception exists about low quality in primary care due to low confidence in the doctors’ knowledge and skills, or difficulties related to communication [9]. Other barriers include cost and time, low perceived need, and fears related to a patient’s medical condition or procedures [10].

The current advancement of medical technologies has placed patients’ views, values, and preferences as central considerations [11]. However, doctors’ or service providers’ values possibly differ from the patients’ values [11]. Hence, patient values should not be defined by other stakeholders in the PHC system, but by the patients’ voices themselves. Patients’ values can be identified in their satisfaction with care, their preferences and priorities, expectations, experiences, and aspects of care important to them [12, 13]. These values can vary widely and are affected by various social, demography, cultural, and health system factors [12, 14]. Taking into account patient values can positively impact healthcare access and delivery, better care continuity and treatment adherence, while minimizing the need for a higher level of care [3, 15]. This qualitative approach in turn can improve health outcomes (decrease preventable morbidities and deaths), and save costs to individuals as well as the health system [13].

Understanding what people value from primary care has also become fundamental for a patient-oriented service provision. This information can help to identify which aspects of PHC are important to patients for further quality improvement. Previous systematic reviews about patient values by Bastemeijer et al. researched the definitions and concepts of patient values and preferences [16]. One study conducted by Mathioudakis et al. also reviewed patient values, but specifically to improve breast cancer screening [17]. There is a lack of information about patient values related to primary health care. This systematic review intended to fill this gap of evidence.

## Methods

### Eligibility criteria

Studies were eligible for inclusion if they:

• were about values from the perspective of and expressed by patients, that is, patients as the participants in the study.
• contained information on values, aspects that patients consider important [18] in primary care.
• used qualitative, quantitative, or mixed-method study designs.

Studies were excluded if they:

• described only views from other stakeholders, such as doctors or healthcare workers on patient values.
• described a context that was outpatient but not PHC, such as in the secondary or tertiary level of care.
• were conference abstracts and briefs since they often contain preliminary findings and insufficient information synthesis [19].

### Search procedure

We sought evidence by systematically searching for original research articles in PubMed and EMBASE (Ovid) (Supplementary file 1) and additional searches in Google Scholar. Articles from January 2009 to May 2020 were included. We identified values and preferences important to patients related to PHC services. Considering values are a complex concept, the inclusion criteria were extended by identifying articles that might implicitly research and explain patient values*.* The search terms were selected from key terms often used in literature for describing patients' values such as satisfaction, important aspects or factors, expectations, priorities, preferences, and experiences [12–14, 16]. We used MeSH terms, keywords, and synonyms to search the articles. The main search terms were "Primary health care", "Patient", "value", and "access" (Supplementary file 1). Search terms were kept broad to capture as many relevant articles within our study objectives.

### Selection process, data extraction, analysis, and synthesis

ABP and JM performed the title, abstract, and full-text screening independently. Through online meetings with all team members, we discussed and resolved disagreements. Due to the variability in methods and data collected in the included studies, meta-analysis was not performed. The data extraction process was guided by the Joanna Briggs Institute (JBI) mixed methods data extraction form [20]. Extracted data were analyzed thematically and synthesized narratively by ABP, RSP, and DW, with input from JM. Firstly, we performed inductive coding to allow concepts and themes to emerge from the data. Coding and concepts were discussed, refined, and finalized with all authors. Secondly, a deductive phase was conducted to categorize the findings. The concepts developed by Bastemeijer et al. [16] were used as an initial guide to classify our findings. This systematic review was presented according to the Preferred Reporting Items for Systematic Reviews and Meta-Analyses (PRISMA) guidelines [21].

### Quality and risk of bias assessment

The quantitative and qualitative studies' quality was assessed using the JBI Critical Appraisal Checklist for Analytical Cross-sectional Studies [17]. This checklist was developed collaboratively, approved by the JBI International Scientific committee, and used in previously published systematic reviews [18–20]. In addition, qualitative studies were assessed using Consolidated Criteria for Reporting Qualitative Studies (COREQ) [22, 23] by ABP and JM. DW and RSP reviewed and gave input to the appraisal results.

## Results

### Study description

**Fig. 1:**
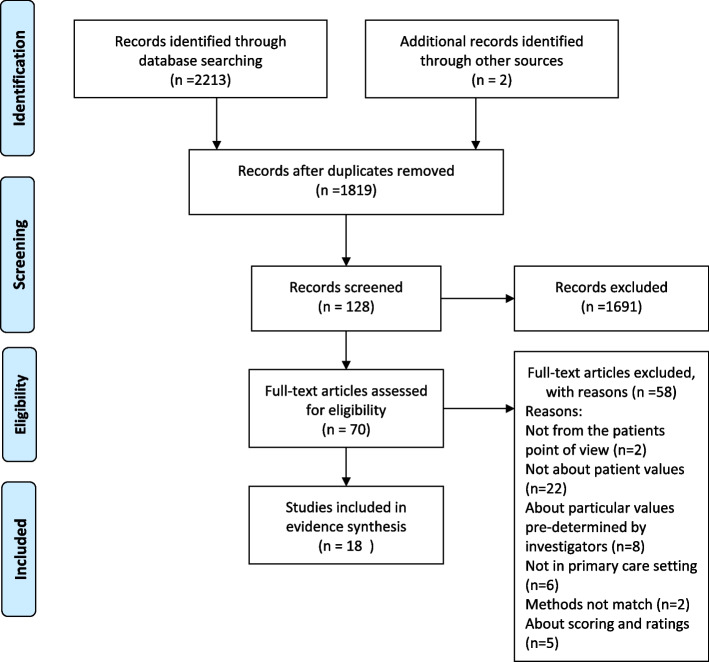
PRISMA flowchart for the included studies [21]

After removing duplicates, a total of 1,819 articles were identified and screened for titles and abstracts, which resulted in 70 articles eligible for full-text screening (Fig. 1). Among them, six full texts were not available. The authors then performed full-text screening for the rest of the articles, resulting in 16 papers and two additional papers from reference searching that met the inclusion criteria. Articles were excluded because of the following reasons: twenty-two articles did not directly reveal patient values; eight papers were about specific aspects and values that had been pre-determined by investigators; six articles focused on other types of care such as emergency; five articles were about scoring and ratings; three studies did not meet the method inclusion criteria (such as using discrete choice experiment), and two studies were not clearly from the patients' point of view. Characteristics of the included studies are described in Tables 1 and 2.

**Table 1:** Characteristics of the included qualitative studies

Characteristics of the included qualitative studies									
First Author	Berkelmans	Ekawati	Marcinowicz	Bjorkman	Chauhan	Pepp	Naidoo	Ross	Artuso
Year	2010	2017	2014	2009	2018	2014	2019	2015	2013
Method/design	Explorative investigation, semi-structured interviews	Interpretative phenomenological analysis	Semi-structured interview; Thematic analysis	Web-based open ended questionnaire	semi-structured interviews	Focus group discussion	Focus group discussion	Praticipatory action research	Descriptive; unstructured interviews, focus groups, and semi-structured interviews
Number of participants	13	23	30	121	19	64	28	85	34
Characteristics of participants: Age	65–91	18–85	65–87	18–60 or older	22–70	Mostly 31–60 years	60 years and above	20–83	38–54
Setting: Country (and category)—based on World Bank	The Netherlands (HIC)	Indonesia (Lower MIC)	Poland (HIC)	Norway (HIC)	Brazil (Upper MIC)	EU (Estonia, Finland, Germany, Hungary, Italy, Lithuania, Spain)—all HIC	South Africa (Upper MIC)	Canada (HIC)	Australia (HIC)
Characteristics of patient participants: Groups	Patients (senior citizens)	Insured citizen	Elderly	Lesbian women	General adult	General adult	Elderly	General adult	Indigenous
Phenomena of interest	Senior citizens value about non-medical aspects of general practitioner	Patients' perspective of a primary care services	Most important aspects of GP behaviour for elderly	Aspects of health care profesionals abilities important to lesbian women	Experiences and perceptions regarding accessibility and quality of primary care	Expectations of patients and professionals on aspects of quality in primary care	Elderly expectations of a primary care	Barriers and enalblers to primary care access for people with mental illness or substance abuse	Factors influencing healthcare utilization

**Table 2:** Characteristics of the included quantitative studies

Characteristics of the included quantitative studies									
First Author	Aldosari	Croker	Droz	Ofei-Dodoo	Kenny	Sebo	Lionis	Mercado	Hirsch
Year	2017	2013	2019	2019	2015	2015	2017	2012	2016
Method/design	Survey	Secondary analysis of English National GP patient survey data (2009)	Survey	Survey	Survey (online)	Questionnaire	Survey	Questionnaire	Questionnaire
Number of participants	37,262	1,476,252	200	2967	2481	1637	219	857	766
Characteristics of participants: Age	16–97	18 and over	Median 55 years (less than 46 to more than 65)	18–114	16—above 70	Mostly (62%) between 25—65 years (Mean 50 years)	18 and above	18 and above	18 and above
Setting: Country (and category)—based on World Bank	Brazil (Upper MIC)	United Kingdom (HIC)	Switzerland (HIC)	Ghana (Lower MIC)	Australia (HIC)	Switzerland (HIC)	Greece (HIC)	United States (HIC)	Germany (HIC)
Characteristics of patient participants: Groups	General adult	General adult	General adult	General adult	General adult	General adult	General adult	General adult	Lesbian women
Phenomena of interest	Patients' satisfaction with dental care in PHC in Brazil	Important aspects of GP consultation	Patients' values of family medicine	Patients' satisfaction on primary care services	Important aspects in choosing a GP	Patients' satisfaction with and expectations from their primary care provider	Patient values of a GP	Important factors when choosing a primary care physician	Expectation towards GP

The selected qualitative studies met between 9 to 10 from 10 JBI criteria, and 18 to 27 from the 32 COREQ criteria (Supplementary file 2). Half of the qualitative studies explicitly explained the interviewers' characteristics, but only one study presented a discussion concerning non-participation. One study had a coding tree description, and the other studies may have put the coding tree directly into themes or subheadings in the main text. Most of the quantitative studies met the critical appraisal criteria checklist. However, the criteria on appropriate exposure measurement, confounding identification, and mitigation strategies did not apply for some of the studies (Supplementary file 2)*.* All authors (ABP, RSP, JM, DW) judged the included studies to be of sufficient quality.

### Patient values of primary care services

Out of eighteen included studies, none came from low-income settings, thirteen originated from high-income countries, and the rest were from middle-income countries (country category according to The World bank) [24]. Nine studies were from European countries. Most respondents aged 18 and above. Vulnerable populations identified within the included studies were elderly, lesbian women, people with mental illness and substance abuse, and ethnic minority groups. No studies were about parent's or children's values.

**Fig. 2:**
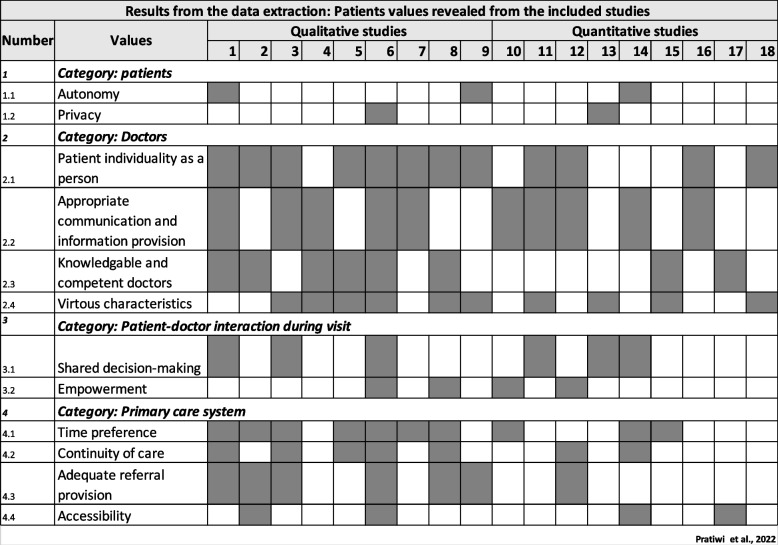
Mapping results from the data extraction. First author and year: (1) Berkelmans et al. (2010) [28], (2) Ekawati et al. (2017) [31], (3) Marcinowicz et al. (2014) [26], (4) Bjorkman et al. (2009) [29], (5) Chauhan et al. (2018), (6) Papp et al. (2014) [35], (7) Naidoo et al. (2019), (8) Ross et al. (2015) [27], (9) Artuso et al. (2013) [33], (10) Aldosari et al. (2017) [38], (11) Croker et al. (2013) [36], (12) Droz et al. (2019) [12], (13) Ofei-Dodoo et al. (2019) [34], (14) Kenny et al. (2015), (15) Sebo et al. (2015) [39], (16) Lionis et al. (2017) [13], (17) Mercado et al. (2012) [40], (18) Hirsch et al. (2016) [37]

**Fig. 3:**
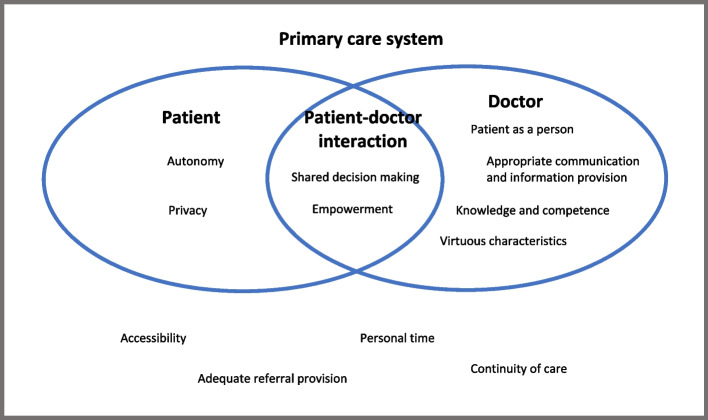
Conceptual framework for patient values regarding primary care

To provide a clearer view at which level the values occur within the PHC system, we categorized the patient values into four groups, as illustrated in Figs. 2 and 3:

- 1. Values related to attributes of the patients themselves (labelled as "patients").
- 2. Values related to their expectations from a primary care physician (labelled as "doctors").
- 3. Values related to the patient-physician interaction (labelled as "patient-physician interaction").
- 4. Values related to the system (labelled as "primary care system").

Values and related quotations from the included qualitative studies are shown in Table 3.

**Table 3:** Quotations from the included qualitative studies

Value	Example quotations
Patient as a person	'As long as you are chronic, they don't care …. They just write down your medication and tell you to go. They don't even look at you. Chronic is just for medication.' (Participant 3, Group 2, 70-year-old female) [25] "She should ask me, how I feel after those drugs" (women aged 79) [26] "If I was a doctor, I would ask – 'how do you feel'? 'What is the matter with you'? But doctors would just repeat the drug prescription ad ask for how long. And good-bye" [26] 'The doctor I'm seeing cannot help me, they refer me to the general doctor. What is the purpose of this doctor I'm seeing?' (Participant 3, Group 3, 68-year-old female) [25] I like the fact that he [family doctor] takes a personal interest in me as his patient. He's a very professional doctor, but he also goes a little bit beyond that, in terms of showing interest. He will want to know how you're doing, and he takes the time to hear something that's not relevant or whatever. You're just saying something that took place in your life. He'll call, on the phone too – 'I haven't seen you this week, is everything ok?' and it's just a matter of checking up. It's wonderful. (Judith, age 67, has a regular provider) [27]
Autonomy	"In my opinion, you should decide yourself which GP to have. …And I have a good GP, I am very pleased to have this GP" (Woman, aged 77) [28]
Communication and information provision	"No matter what I wanted to bring up;……..she switched to saying that being lesbian had to be very hard….I changed doctors" [29]
Virtuous characteristics	"On the lighter side there's also really, really good ones. They'll say, 'You know what dear? I can see you're addicted to drugs or opiates but hey, you know what? I'm gonna try to help.'….The ones that are non- judgmental; they take your breath away." (Francesca, age 52, has a regular provider) [27] "I can't access anybody…There is no access. I don't have money. If I had money… if I was, you know, a politician, there'd be lots of access. If I was working a straight job, there'd be lots of access. But because I'm an ex-convict, whose got mental health and addiction problems, there's nothing for me." (Garret, age 47, does not have a regular provider) [27] "There was a doctor… He was so rude to me. I told my husband I would never come back to see this doctor again. He told me: 'You are a mess!' screaming at me, 'You're going to lose your leg! I said to him 'Hey this is not the way you should talk to me' but he kept going on [Participant 2, Black][30] "I think if I were white people would treat me better. They would be nicer and talk to me longer [Participant 58, Brown] [30] "I only gained access because I had a friend who started working here who got it for me because I came here and I couldn't get [an appointment] [Participant 98, Yellow] [30] "The ones who have easy access are the ones who know somebody in the system" [Particpant 86, White] [30] "I tried even to get blood tests or something… they go, 'What? You don't have a health card so you're gonna have to pay cash for it.' So then never mind, 'See you later' and then I leave." (James, age 53, has a regular provider) [27]
Knowledgeable and competent	"I said 'I have DID [dissociative identity disorder]' and she [health care provider] said, 'What's that?' And I [told] her and she stopped the interview right there and said 'We don't deal with people like you here'….So I just left and started crying, and walked home.'" (Lanette, age 34, has a regular provider) [27]
Shared decision-making	"We were thinking together, what to do in my situation; it was a relaxed conversation" (Man, aged 75) [26] "An open mind and atmosphere in which anything can be discussed and in which, for example, any different ideas that patients may have are taken seriously. Not one in which the doctor says in a superior manner: Well, we've got the picture now" (Man, aged 90) [28]
Empowerment	"[The doctor] didn't lecture me. He didn't make me feel bad. He just said 'Well, I know you're a smart girl' and he kind of gave me credit for knowing better. And he said 'this is what I want you to do—and if it's not working out, I want you to call me. Either way, I want to see you back here in 6 months and we'll take it from there.' And in 6 months, I had actually stopped smoking." (Keeya, age 44, has a regular provider) [27]
Preferences for time	"If in Puskesmas, I need to queue for a long time. I have to queue before here and there. But, I need to go working, so I decide to leave the Puskesmas and go to private hospital" (Participant 9, I. 38–39) [31] "Sometimes, I make an appointment, and sometimes I just go to the drop-in because making an appointment is a sense of hassle. You have to wait 2 weeks, 3 weeks, things like that." (Norine, age 33, has a regular provider) [27] I can make appointments to see [a primary care provider at the community health centre where he was interviewed] but people with mental issues and addictions– I have a very severe addiction. I'm a crack addict. For me to sit here for a half an hour right now, it's killing me. (Barry, age 51, does not have a regular provider) [27] 'The biggest worry is the queue … sometimes you spend the whole day here.' (Participant 4, Group 1, 66-year-old female) [25] 'All of us have to wait, and they can tell us to come back next day.' (Participant 1, Group 2, 70-year-old female) [25] 'We are in our 70 s. Imagine … paying all the taxi fare, coming back the next day.' (Participant 2, Group 1, 72-year-old female) [25] "The time it takes to get an appointment is the biggest problem. It is too long… At 7 they distribute the numbers so I need to arrive at 5 to be among the first 20 [patients] otherwise I don't get it. There are many people here. They would not be here if they didn't need it [Participant 2, Black] [30]
Continuity of care	"Because he has been our doctor for so many years. I Don't have to say much to him, really. He knows me inside out" [28] "If you go to your own family doctor—unless he's very open-minded–you'll be shamed out of there, you know. You certainly can't go to a walk-in clinic and mention [substance use] because they call it drug- seeking." (Arlene, age 54, does not have a regular provider) [27]
Preferences for referral	"He's concerned about my well-being, mental health and my physical health. Mentally – he's the one they referred me to [tertiary care mental health hospital]. If it's something that he sees that's outside of his scope as an MD [family doctor] – that's what I like about him. He looked for other sources, in a more specialized area. Here I could see a psychiatrist or someone practicing psychiatric medicine here. And that's out of his scope. And he's humble enough to know that and refer you to someone else. Because he wants the best for me, I think." (Judith, age 67, has a regular provider) [27] "I like the fact that [Community Health Centre] is a one stop shop. So, you can see a nurse. You can see a lab tech. You can see a physician. They'll make referrals within their system." (John, age 55, has a regular provider) [27]

### Patients 

From our analysis, there are two values closely associated with the patient: autonomy and privacy.

#### Autonomy

We found that for patients, autonomy means being given choices. The study by Kenny et al. found that the opportunity to choose which General Practitioner (GP) to see was regarded as an essential aspect by more than 80% of Australian adults [32]. Vulnerable groups prioritized more autonomy, because they may feel the lack of it. Berkelmans et al. found that autonomy is a need for senior citizens in the Netherlands. In their study, some patients who got a home visit from doctors would prefer to be given a choice to visit the GP at the practice [28]. Indigenous respondents in Australia accentuate the need for autonomy. They were provided with less information, have inadequate ability to understand medically related information, and felt they were left without options [33]. An empowering respondent illustrated this value: *"Giving people the power to be able to say, 'Well, we want this' and then resource those ideas….."* [33]*.*

#### Privacy

We could not find any explicit description about the specific aspects patients hold important about privacy. One recent survey found that patients’ experience that their privacy is protected during visits, increases their satisfaction by 1.34 (95% CI: 1.10 – 1.63) [34]. In this research, the question asked about privacy was “What was the way the health services ensured that you could talk privately to providers?”. Papp et al. (2014) found that patient privacy and information confidentiality during the PHC visit is essential but rarely expressed by patients from the EU nations [35]. This focus group discussion (FGD) study categorized privacy during the primary care visits and patients’ information as part of patient-centered care.

### Doctors

We found that patient values were predominantly associated with the values of the GP. Patients hold high expectations towards their GPs even though the system and regulation also contribute to patients’ experience. Some values related to the GP were especially crucial, to such an extent that it would be intolerable if violated by the physician. Some studies found that patients would rather change providers if their personal values are breached. However, in practice the GPs might not be fully aware of this situation.

#### Patient as a person

Eight studies revealed the value of treating patients as a person [12, 13, 25–27, 30, 33, 35], and in complement, there were six studies that emphasized the importance of being taken seriously [12, 13, 26, 28, 31, 36, 37]. Patients would rather be seen and treated as a whole person, meaning as an individual with needs concerning their medical conditions, than to be merely seen as a medical case. Patients perceive that their complaints, illness, and medical situation often become the doctor's sole focus without adequately considering their psycho-social needs. To be seen as an individual also meant to be taken seriously by the doctor, being asked about feelings and concerns.

Studies in this review emphasized the value of being taken seriously [12, 13, 26, 28, 31, 36, 37]. One possible way to interpret this value would be that the patient wants to be seen as a person whose health problems should be dealt with by having their medical conditions assessed and treated holistically by the physician. In the findings from a United Kingdom (UK) study, the aspect "GP that takes patients problems seriously" was ranked as the most critical component in GP consultation, consistent across different genders, ethnicities, and age groups, but no further explanation was provided about what they meant. Having their problems seriously addressed increases patients' confidence and trust in their GP by three times [36].

Among specific population groups, elderly with chronic diseases in South Africa experienced an uncomfortable disease-centered form of care where the GPs focus only on providing medication [25]. An elderly patient visiting the geriatric section at primary care felt frustration and ignorance when they were immediately referred to another doctor without proper explanation [25]. Furthermore, individuals with mental illness or substance abuse often have complex and interrelated physical, medical, and psychosocial needs that are crucial to be seen as a whole person for their well-being [27].

#### Appropriate communication and information provision

The doctors’ excellence in communication and information provision was an important value by studies among Australians [32], elderly in Poland [26], dental patients in Brazil [38], UK patients [36], and patients in Switzerland [12]. Adequate doctors’ explanation was also ranked as the third most important aspect by UK patients, notably ranked more critical among the subgroup age 35–64 white people, but rated lower among elderly non-white [36]. A study conducted in Switzerland showed that communication is considered the most important value of family medicine. Getting a clear understanding of what their GP explains was regarded as very important by 70% of respondents [12]. In contrast, a small number of elderly patients in The Netherlands prefer less information or would instead obtain information independently from other sources such as the Internet [28].

Patients suggest that communication and information provision affect the quality of care they receive. The willingness of doctors to explain and provide information affects patients’ satisfaction with care [26, 32, 36, 38]. For some patients, the value of communication and information provision is fundamental so that a patient may decide to go to another service if this value was not met. Inappropriate languages and expressions can be perceived as offensive by patients. For example, lesbian women who felt that the physician responded improperly to their situation decided to see another doctor [29]. The situation might occur due to the doctor’s limited understanding of the lesbian patients’ context. Sometimes, patients are left confused with the information provided by the GP or thinking that the doctor’s explanation is sometimes insufficient. Some patients have encountered doctors who barely explain their health conditions but instead jump directly to giving prescriptions [25].

Listening to patients is also highly valued as part of good communication skills [12, 13, 28, 32, 35]. Being listened to also meant being asked back about details, since patients may have difficulties even saying anything due to their limited understanding of diseases [26]. According to a survey in Greece, patients want to be given a chance to ask questions [13].

#### Knowledgeable and competent doctors

In eight of the studies, patients value the knowledge, skills, and competence of GPs [27–31, 35, 39, 40]. Having sufficient knowledge and skills is perceived to determine the doctor's ability to treat patients adequately and deliver relevant information [28]. Knowledge does not only refer to medical comprehension but also about contextual features inseparable to the patients’ life and well-being. The certified doctor was also considered as a competent doctor as shown in two studies about preference for seeing a certified GP [39, 40].

Some patients in Indonesia go to PHC only for minor illness. They perceive the GP to have insufficient knowledge and skills to treat more serious illness [31]. A study on patients with mental disorders explained that the feeling of being offended and mistreated may make them decide to leave the GP and not come back [27]. Similarly, lesbian women noted that GPs commonly lack awareness of the lesbian context, and perceive patients generally as heterosexual. Hence, doctors may relate lesbian patients’ medical condition inappropriately to their relationship preference [29].

#### Virtuous characteristics

Physicians in PHC are expected to uphold and demonstrate virtues, including attitudes of non-discrimination, inclusivity. Six studies [27, 29, 30, 33, 35–37] emphasized the importance of non-discriminatory and non-judgmental treatment from GPs to facilitate a favorable care experience. In one European study, none of the participants reported being discriminated against in primary care [35]. However, in other parts of the world, patients feel that they did not receive satisfactory treatment because of racial, skin color, and socio-economic-status-related discrimination [27, 30]. In addition, some individuals received privileged access due to pre-existing connections with PHC staff. Patients experiencing discrimination in Brazil were unwilling to see the same physician again [30].

Inclusiveness is considered an essential value, as reflected in studies among specific groups, including lesbian women, the elderly, indigenous communities, and patients with mental illness [27, 29, 39]. For mental health patients who have complex social circumstances, a non-inclusive service provision may increase the risk to forgo the care altogether [27]. An integrated and inclusive care provision is needed to fulfil their health needs.

The virtues were further expanded to attributes that have been commonly associated with ideal physicians such as being empathetic and respectful [29, 30, 34], open-minded [26], friendly [26, 35], accepting [29], understanding [29], open [29], supportive [29], attentive [30, 35], as well as comforting [30]. These attributes have been shown to contribute to increasing patient satisfaction, as found by a study from Ghana [34].

#### Patient doctor interaction during visit

Empowerment and shared-decision making emerged as essential features from the patient-doctor interaction.

##### Shared decision-making

Patients valued being asked and involved in decisions concerning their medical conditions and treatment [26, 28, 32, 34–36]. Shared decision-making for patients meant having their views taken into deliberation, an open discussion, and avoiding paternalistic decisions. Shared decision-making was perceived to lead to better treatment adherence.

A shared decision is highly valued by patients in Europe [26, 28, 35]. Among the general population in the UK, shared decision-making is ranked as the fourth most important aspect of a primary care doctor. It is ranked the second most crucial aspect by the white elderly population aged above 65 years but ranked lower notably by non-white young people below 35 years old [36]. In this study, there was no difference in the rank of shared decision-making among different genders [36]. The study in Ghana found that the involvement of patients in the decision-making process increases their satisfaction with primary care by 1.34 times [34].

##### Empowerment

Patients emphasized the importance of being empowered by their GPs [12, 27, 35, 38]. Patients who have a good relationship and support from their PHC provider will feel confident and empowered to care for their own well-being [27]. Although considered vital in care, patient activation as described by adherence to the agreed plan and fulfilling scheduled appointments are valued relatively less in Switzerland. Only half of the respondents regarded it as very important [12].

### Primary care system

#### Time preference

Having a visit to primary care services often means a trade-off to other activities, including work. People prefer shorter waiting times inside the clinic and brief queues to get appointments, yet patients also regarded sufficient consultation time as crucial [26, 32, 38]. Patients expected a longer consultation time with the PHC provider than the current allocated time, including sufficient time to get the explanation of their medical condition [35]. Among dental primary care users in Brazil, patients receiving enough time for treatment were more satisfied [38].

This situation causes a trade-off to choose specific patients values for the primary care system, where it is impossible to accommodate all. In European countries, concerns in queuing are more directed towards the waiting time from calling or getting in contact with the clinic until getting the appointment schedule. In middle-income countries such as Indonesia, Brazil, and South Africa, the time spent for queuing at primary care can be problematic, because the patients have to show up physically and wait.

The acceptable waiting time in clinics varies between studies. For the elderly and patients with addiction, waiting at the clinic for more than a half hour was considered to be long [27, 28]. In Brazil, some primary care patients needed to show up early in the morning to queue [30]. Long waiting times also result in higher opportunity for community acquired infection as well as increased dissatisfaction and indirect costs. In Indonesia, lengthy waiting times increases the risk of patients leaving the PHC and changing to a private hospital because the opportunity costs for working people will be high [31]. Similarly, in South Africa, people can spend their whole day queuing or, worse, having to come back the next day, which entails additional costs on transportation [25].

Acceptable waiting time in terms of days to get appointments varies, ranging from zero days (getting appointment immediately at arrival) to two weeks [27, 28, 30, 35, 38, 39]. In some countries, any waiting time that impedes access to care is unacceptable. Meanwhile in Finland, one week of waiting for an appointment schedule is considered acceptable [35]. A study from Brazil found that patients accepted for dental treatment upon arrival were more satisfied than those who were scheduled [38]. Getting immediate appointments was also preferred by patients with mental health conditions. In the usual situation in one study, they may have to wait two to three weeks [27].

In terms of age, young patients were reported to have less tolerance for lengthy waiting time at the clinic than waiting for an appointment, while the elderly were more demanding in expecting to meet the doctor on the same day [39]. Regardless of age, most respondents considered a waiting period of up to two weeks appropriate and tolerable [39].

#### Continuity of care

Patients value the continuity of care by seeing the same doctor [12, 26–28, 30, 32, 35]. The doctor is expected to know the patients’ personal as well as their medical history. This aspect makes the patients feel familiar and comfortable to visit the primary care since they do not have to explain their condition again for the subsequent visits. Seeing the same doctor also means that the patient and the doctor can build a more personal relationship, resulting in the patient's trust and confidence in the doctor. It is regarded as important by individuals in seven EU countries as reflected by one respondent [35]:

*‘“It is important that one doctor sees the whole process of the illness. So, the patient should not tell another doctor the whole case history again and again”. (Hungarian patient)'* [35]

Continuity of care was particularly valued by patients with medical conditions needing multiple visits. This value was described as knowing the GP that patients will meet which was regarded as very important by more than 50% of Swiss respondents, and more importantly, among patients with chronic disease [12]. The importance of continuity of care for people with mental health issues was also divulged from a study in Canada that indicated as many as 80% of participants who see the same GP regularly have a good relationship with their health care provider [27].

#### Adequate referral provision

Patients also valued referral provision [12, 26–28, 31, 33, 35] because sometimes they encountered difficulties in obtaining an appropriate referral. Some perceive the gatekeeping role of primary care, and practitioners’ reluctance hinder people from obtaining a referral. Others would expect the humility of the practitioners they see to offer a referral immediately if the medical condition is outside of their expertise [27]. Patients prefer direct access instead of going to the GP only to get the specialist's referral letter [35]. GPs’ decision to refer to a specialist is considered very important by almost 70%, and the second most substantial value of family medicine by respondents in Switzerland [12]. In a study from Poland, geriatric patients perceived that doctors were reluctant to provide a referral to a specialist [26], as illustrated by one respondent in the statement below:

‘‘‘Somehow doctor was not very eager to give a referral for tests’’ (Woman, aged 79).’

This issue that patients have to go back and forth between referrals is also seen in the back referral process (referral from a higher level of care back to the primary care). When patients are referred to the specialist and then back to primary care, the GP will convey that it is beyond their competence and capacity [35]. In one study, aboriginal patients with cardiac conditions were commonly left without follow-up care once they had finished with treatments at the hospital and returned to their community [33].

#### Accessibility

Ease of access is another important element that patients consider highly when choosing PHC that emerged from the data. Services that can be easily reached through phone calls and are closer in proximity are preferable [31, 31, 32, 35, 40]. Similarly, geographical access and time needed to reach PHC were influential aspects for patients in Europe [35].

In terms of financial accessibility, out-of-pocket payments may impede accessibility of PHC. Patients in Germany expect no charge for access to primary care, even for preventive care, because not everyone can pay [35]. Costs are closely linked to the primary care services that the health system implements, and they have been brought up as a significant factor in choosing GPs by patients in Australia [32].

### Description of factors affecting values

Although the essential values of primary care arising from the included studies were mainly similar, different social determinants such as age, gender, ethnicity, disease, and rurality might influence patients' values regarding PHC. Findings from the studies varied, particularly appearing from the analysis in some quantitative studies. According to two studies, the most important values do not differ between gender, although women assigned higher values [12, 32]. Specifically, for vulnerable groups, the value of non-discriminatory services should be seen as a top priority, because it could potentially influence their decision to visit a particular PHC [27, 29, 30, 33, 36, 37]. In a survey in Switzerland, participants with chronic diseases valued the aspect of being seen as a person and continuity of care higher than people without chronic illness with OR 2.21 and 1.92, respectively [12]. In contrast, the study in Australia found that having a chronic disease and rurality do not differ in all their modelling in essential aspects of GP [32]. However, age and GP visit frequency did. According to Sebo et al., elderly and patients at large practices are more stringent [39].

## Discussion

Our review sought to understand the vital aspects of health care services that patients value regarding PHC. Our findings describe that values in primary care vary; some values influence outcome measures such as patient satisfaction, trust, and utilization. Concerning patients’ experience, privacy and autonomy are crucial. Related to the doctor, patients accentuate the importance of being seen as a whole person and expect doctors to have good knowledge and competence in medical and non-medical aspects. Patients also valued doctors’ virtuous traits. Shared decision-making and empowerment were critical in the interaction between doctor and patient. Other elements that patients value specifically related to the primary care system arrangement were waiting time, being able to see the same doctor, being given a referral, and accessible primary care.

### Virtuous characteristic of doctors and patient as a person

Values related to the general practitioner were paramount and apparent in most studies from high- and middle-income countries. This may reflect that even in a good PHC system, patients place high expectations on their doctor's interpersonal skills. Patients value primary care doctors who have not only medical-related competence but also virtuous characteristics and can address their patients' as a person, as in findings from previous studies [41–43]. A study from the UK found the most critical component of general practitioner consultation was having their problems addressed seriously, which increases trust and confidence in GP. Findings from other countries also emphasized this value. The picture of an ideal physician may not always be realistic. However, it reflects the need for primary care providers to pay attention to tailored care to address patients' specific needs.

### Value prioritized by vulnerable subgroups

In healthcare and daily life, vulnerable groups experience stigma and discrimination daily outside healthcare, from stigma related to the socio-economy, and criminalization, to unsettled housing, which can become notable barriers to accessing PHC. Our study highlights that vulnerable subgroups may prioritize a particular value regarding PHC differently than general adult patients. For example, the elderly and indigenous accentuated their need for autonomy. In contrast, lesbians and patients with mental disorders prioritized inclusivity and understanding their psycho-social context. In Europe, patients don't feel discriminated against in PHC [27]. However, this is not the case in other parts of the world, including studies from Brazil and Canada. Hence, it is crucial to operationalize an inclusive care provision at PHC with patients' views.

### Continuity of care and referral

Seven studies from different countries valued the continuity of care, which is unique for PHC. For patients needing multiple visits or those with chronic illness, continuity of care is regarded as relatively more critical, similar to findings from previous studies [44, 45]. Continuity of care impacts health outcomes and may lead to decreased mortality in primary and secondary care [45]. Through this continuity of care, patients experience the benefit in that the doctor remembers patients’ information, so they do not have to repeat the same story. This can save the uncomfortable process for the patient of explaining their medical condition and establish trust and a good relationship [12, 27, 32]. PHC may operationalize continuity of care differently, either at the healthcare facility or doctor level. In the findings, patients value the latter.

Furthermore, referral provision is also unique to PHC. Our findings highlight the importance of the PHC doctor's decision to provide a referral. However, some patients perceived reluctance and experienced difficulties related to the referral. Referral provision-related policies may restrict doctors from providing referrals [4, 8]. Due to limited understanding of this gatekeeping system, patients may demand that GPs give referrals to a higher level of care [31]. Some patients perceive GP's gatekeeping role as a barrier to reaching specialist care [35], creating a dilemma for the doctor.

### Privacy and autonomy

We noted that although privacy protection increased patient satisfaction [34], it is rarely explicitly framed. Privacy protection is closely related to patients' trust and care-seeking should be possible without any privacy infringement [46–49]. Attention to patient privacy should be reflected at least in two aspects: during the visit and on patients' information. Research on privacy in healthcare recently leaned towards discussing electronic patient records and online data protection [49–53]. Despite being equally important, there was little discussion of the other dimensions of privacy, such as how the patient felt and experienced their privacy. Privacy during PHC visits might not be an issue in some countries, but this gap suggests there is scarce evidence about how and which aspects of privacy patients value in primary care.

Autonomy in primary care can be particularly challenging for vulnerable groups. Respecting a patient's autonomy can be, at a certain point, challenging when in conflict with the value of evidence-based medicine [54], for example, for doctors to find the balance between their medical knowledge and patients' wishes. Research on patients with chronic illness found that autonomy is recognized as a value underlying patients' demand for quality services, while paternalism and ‘knowing better’ can reflect a lack of recognition of patients' autonomy [55]. Autonomy is also closely related to the second theme; doctor-patient interaction relates to the values of shared-decision making and empowerment.

According to the IOM and Picker institute, values should guide clinical decisions [56, 57]. Patient values identified in our study can be used as a basis for PHC aiming at patient-centered care. This kind of patient-centered approach could be implemented, for example, into a guideline that incorporates patients' values [58, 59].

### Strengths and limitations

To our knowledge, this is the first systematic review on patient values regarding primary care. We used rigorous methods through the search steps and assessment of the articles. We used the most relevant studies, suggesting that the values reflected are related to the current situation. The studies included different populations, and countries, thus capturing various settings and circumstances.

A limitation of this study is that it only included studies published in English. Related to the included studies, elaboration and description of particular values were often unavailable. As a result, we could not compare possible different meanings concerning a specific value in different studies.

## Conclusions

This study provides insight into aspects and values from the patients' perspective that may affect their decision towards seeking care at a PHC. Countries that want to set a primary care system as the basis for universal health coverage should prioritize and consider the aspects patients think are important about PHC. Patients value the interpersonal and virtuous characteristics of a PHC doctor, which demands particular attention. Patients values in primary care were mainly related to the doctor or interaction during clinical encounters, which is unique to PHC. Although we assumed that privacy might be an essential value, patients rarely expressed it in the studies. Different subgroups of patients may prioritize values differently. Further identification of priority values concerning patient characteristics can inform patient-centered service provision. Continuity of care, good referral provision, and accessibility were values related to the PHC system that need careful attention. There were no studies from low-income countries, nor about parents' or children's specific values. Further research may need to concentrate on these two blind spots.

## Supplementary Information

**Additional file 1: Supplementary file 1.** Main search strategy.

**Additional file 2: Supplementary file 2.** Critical appraisal for the articles.

**Additional file 3: Supplementary file 3.** PRISMA 2020 checklist.

## Data Availability

Data from included articles in this study are available in the reference for download. Data generated during the analysis are available in the main text and supplementary materials.

## Abbreviations

PHC: Primary Health Care
JBI: Joanna Briggs Institute
FGD: Focus group discussion
