# Supplementary material for: Patients values regarding primary health care: a systematic review of qualitative and quantitative evidence
Source: BMC Health Serv Res. 2023 Apr 25;23:400. doi: 10.1186/s12913-023-09394-8 (PMC10131468; doi:10.1186/s12913-023-09394-8)
Supplement: Supplementary file 1 — Additional file 1: Supplementary file 1. Main search strategy. [file 12913_2023_9394_MOESM1_ESM.docx]

**Supplementary file 1.** Main search strategy

| # | Searches |
| --- | --- |
| 1 | exp *primary health care/ or *family medicine/ or *general practitioner/ or *health center/ or *community care/ or (family medicine or family practice or family healthcare or family health care or primary care or primary healthcare or primary health care or general practice or family physician*).ti,ab,kw. |
| 2 | patient care/ or (patient* and (value* or need* or participation or perception or expectation* or preference* or communication or continuity or coordination)).ti,ab,kw. or (patient priorities or disparities).ti,ab,kw. |
| 3 | (gatekeep* or partnership* or interpersonal relationship* or primary care appointment* or quality of care or quality patient-centered care or time for care).ti,ab,kw. |
| 4 | health care delivery/ or (access* or service*).ti,ab,kw. |
| 5 | 1 and 2 and 3 and 4 |
| 6 | editorial/ or letter/ or case report/ or exp drug/ or exp practice guideline/ or (letter or case report* or comment or editorial).ti. or (therap* or intervention*).ti,ab,kw. |
| 7 | 5 not 6 |
| 8 | limit 7 to conference abstract status |
| 9 | 7 not 8 |
| 10 | limit 9 to english language |
| 11 | limit 10 to yr="2009 -Current" |

*What are the important values to patients for a primary care service?*

5-2-2020:

| Databases: |  |  |
| --- | --- | --- |
| PubMed/Medline, Embase (Ovid) | Before deduplication | After deduplication |
| Total | 2213 | 1839 |

*Searches before deduplication:*

PubMed/Medline:

1225 hits:

("Family Practice"[MAJR] OR "Primary Health Care"[MAJR] OR "Physicians, Primary Care"[MAJR] OR "Physicians, Family"[MAJR] OR "General Practice"[MAJR] OR "Community Health Centers"[MAJR:NoExp] OR "Community Health Services"[MAJR:NoExp] OR family medicine[tiab] OR family practice[tiab] OR family healthcare[tiab] OR family health care[tiab] OR primary care[tiab] OR primary healthcare[tiab] OR primary health care[tiab] OR general practice[tiab] OR family physician*[tiab])

AND

("Continuity of Patient Care"[Mesh] OR (patient*[tiab] AND (value*[tiab] OR need*[tiab] OR participation[tiab] OR perception[tiab] OR expectation*[tiab] OR preference*[tiab] OR communication[tiab] OR continuity[tiab] OR coordination[tiab])) OR patient priorities[tiab] OR disparities[tiab])

AND

(gatekeep*[tiab] OR partnership*[tiab] OR interpersonal relationship*[tiab] OR primary care appointment*[tiab] OR quality of care[tiab] OR quality patient-centered care[tiab] OR time for care[tiab])

AND

("Health Services Accessibility"[Mesh] OR access*[tiab] OR service*[tiab])

AND

(english[Language])

NOT

("Letter"[Publication Type] OR "Editorial"[Publication Type] OR "Comment"[Publication Type] OR "Guideline" [Publication Type] OR "Case Reports" [Publication Type] OR "Therapeutics"[Mesh] OR "Chemicals and Drugs Category"[Mesh] OR "Diseases Category"[Mesh] OR therap*[tiab] OR intervention*[tiab] OR letter[ti] OR editorial[ti] OR case report[ti])

AND

("2009/01/01"[Date - Publication] : "2020/02/05"[Date - Publication])
