# Supplementary material for: Patients values regarding primary health care: a systematic review of qualitative and quantitative evidence
Source: BMC Health Serv Res. 2023 Apr 25;23:400. doi: 10.1186/s12913-023-09394-8 (PMC10131468; doi:10.1186/s12913-023-09394-8)
Supplement: Supplementary file 2 — Additional file 2: Supplementary file 2. Critical appraisal for the articles. [file 12913_2023_9394_MOESM2_ESM.docx]

Supplementary file to Pratiwi et al. (2023) - Patients values regarding primary health care: a systematic review of qualitative and quantitative evidence

**Supplementary 2.** Critical appraisal for the articles

| **Critical appraisal for included quantitative studies** | | | | | | | | | | | |
| --- | --- | --- | --- | --- | --- | --- | --- | --- | --- | --- | --- |
| JBI Critical Appraisal checklist for Analytical cross sectional studies [19] | | | | | | | | | | | |
| For each criteria we chose the following: Yes / No / Unclear / Not Applicable (NA). | | | | | | | | | | | |
| **Review questions** | | | | | | | | | | | |
| **First Author** | **Year** | **1. Clear inclusion criteria** | **2. Detail subjects and setting description** | **3. Valid and reliable exposure measurement** | **4. Objective/ standard measurement criteria** | **5. Confounding identification** | **6. Strategies to deal with confounders** | **7. Valid and reliable outcomes measures** | **8. Appropriate statistical analyses** | **9. Overall appraisal (include/ exclude** | **10. Reasons for exclusion** |
| Aldosari | 2017 | Yes | Yes | Yes | Yes | Yes | Yes | Yes | Yes | Include | NA |
| Croker | 2013 | Yes | Yes | Yes | Yes | Yes | Yes | Yes | Yes | Include | NA |
| Droz | 2019 | Yes | Yes | Yes | Unclear | NA | NA | Yes | Yes | Include | NA |
| Ofei-Dodoo | 2019 | Yes | Yes | Yes | Yes | Yes | Yes | Yes | Yes | Include | NA |
| Kenny | 2015 | Yes | Yes | NA | Yes | NA | NA | Yes | Yes | Include | NA |
| Sebo | 2015 | Yes | Yes | Yes | Yes | Yes | Yes | Yes | Yes | Include | NA |
| Lionis | 2017 | Yes | Yes | Yes | Yes | No | No | Yes | Yes | Include | NA |
| Mercado | 2012 | Yes | Yes | Yes | Yes | Yes | No | Yes | Yes | Include | NA |
| Hirsch | 2016 | Yes | Yes | NA | Yes | NA | NA | Yes | Yes | Include | NA |

The articles were appraised using the Joanna Briggs Institute (JBI) critical appraisal checklist [19], and included if there were no serious concerns about the quality and approved by all authors.

| **Critical appraisal for included qualitative studies** | | | | | | | | | | | | |
| --- | --- | --- | --- | --- | --- | --- | --- | --- | --- | --- | --- | --- |
| JBI critical appraisal checklist for qualitative studies | | | | | | | | | | | | |
| For each criteria we chose the following: Yes / No / Unclear / Not Applicable (NA) | | | | | | | | | | | | |
| **Review questions** | | | | | | | | | | | | |
| **First author** | **Year** | **Aims clearly stated** | **Appropriate qualitative method** | **Appropriate study design** | **Appropriate participant recruitment** | **Collected data adressing the issue** | **Consideration of researcher-participants relationship** | **Consideration of ethical issues** | **Rigorous data analysis** | **Clear findings** | **Valueable study** | **Overall apprisal (Include/exclude)** |
| Berkelmans | 2010 | Yes | Yes | Yes | Yes | Yes | Yes | Unclear | Yes | Yes | Yes | Include |
| Ekawati | 2017 | Yes | Yes | Yes | Yes | Yes | Yes | Yes | Unclear | Yes | Yes | Include |
| Marcinowicz | 2014 | Yes | Yes | Yes | Yes | Yes | Unclear | Yes | Yes | Yes | Yes | Include |
| Bjorkman | 2009 | Yes | Yes | Yes | Yes | Yes | No | Yes | Yes | Yes | Yes | Include |
| Chauhan | 2018 | Yes | Yes | Yes | Yes | Yes | Yes | Yes | Yes | Yes | Yes | Include |
| Papp | 2014 | Yes | Yes | Yes | Yes | Yes | Yes | Yes | Yes | Yes | Yes | Include |
| Naidoo | 2019 | Yes | Yes | Yes | Yes | Yes | Yes | Yes | Yes | Yes | Yes | Include |
| Ross | 2015 | Yes | Yes | Yes | Yes | Yes | Yes | Yes | Yes | Yes | Yes | Include |
| Artuso | 2013 | Yes | Yes | Yes | Yes | Yes | Yes | Yes | Yes | Yes | Yes | Include |

The articles were appraised using the Joanna Briggs Institute (JBI) critical appraisal checklist [19], and included if there were no serious concerns about the quality and approved by all authors.

| **First author** | **Year** | **Domain 1: Research team and reflexivity** | | | | | | | |
| --- | --- | --- | --- | --- | --- | --- | --- | --- | --- |
|  |  | **Personal characteristics** | | | | | **Relationship with participants** | | |
|  |  | **1** | **2** | **3** | **4** | **5** | **6** | **7** | **8** |
| Berkelmans | 2010 | Yes | Yes | Yes | No | No | No | Yes | No |
| Ekawati | 2017 | Yes | Yes | Yes | No | No | No | Yes | Yes |
| Marcinowicz | 2014 | Yes | Yes | Yes | No | Yes | Yes | Yes | No |
| Bjorkman | 2009 | Yes | Yes | Yes | No | No | No | No | No |
| Chauhan | 2018 | Yes | Yes | Yes | No | Yes | Yes | Yes | Yes |
| Pepp | 2014 | Yes | Yes | Yes | No | No | No | Yes | Yes |
| Naidoo | 2019 | No | Yes | Yes | No | No | No | No | No |
| Ross | 2015 | Yes | Yes | Yes | No | No | Yes | Yes | No |
| Artuso | 2013 | Yes | Yes | Yes | No | No | Yes | Yes | Yes |

Consolidated Criteria for Reporting Qualitative Studies (COREQ) [21]. Domain 1: research team and reflexivity, the criteria are (1) interviewer, (2) credentials, (3) occupation, (4) gender, (5) experience, (6) relation established, (7) participants know the interviewer, (8) interviewer characteristics.

| **First author** | **Year** | **Domain 2: study design** | | | | | | | | | | | | | | |
| --- | --- | --- | --- | --- | --- | --- | --- | --- | --- | --- | --- | --- | --- | --- | --- | --- |
|  |  | **Theoretical framework** | **Participant selection** | | | | **Setting** | | | **Data collection** | | | | | | |
|  |  | **9** | **10** | **11** | **12** | **13** | **14** | **15** | **16** | **17** | **18** | **19** | **20** | **21** | **22** | **23** |
| Berkelmans | 2010 | Yes | Yes | Yes | Yes | Yes | Yes | No | Yes | No | No | Yes | No | No | Yes | No |
| Ekawati | 2017 | Yes | Yes | Yes | Yes | No | Yes | No | Yes | No | No | Yes | No | Yes | Yes | No |
| Marcinowicz | 2014 | Yes | Yes | Yes | Yes | No | Yes | No | Yes | Yes | No | Yes | No | Yes | Yes | No |
| Bjorkman | 2009 | Yes | Yes | Yes | Yes | No | Yes | No | Yes | Yes | No | Yes | No | Yes | Yes | No |
| Chauhan | 2018 | Yes | Yes | Yes | Yes | No | Yes | Yes | Yes | Yes | No | Yes | Yes | Yes | Yes | No |
| Pepp | 2014 | Yes | Yes | Yes | Yes | No | Yes | Yes | Yes | Yes | No | Yes | Yes | Yes | No | No |
| Naidoo | 2019 | Yes | Yes | Yes | Yes | No | Yes | No | Yes | Yes | No | Yes | Yes | Yes | Yes | No |
| Ross | 2015 | Yes | Yes | Yes | Yes | No | Yes | No | Yes | Yes | No | Yes | Yes | Yes | Yes | No |
| Artuso | 2013 | Yes | Yes | Yes | Yes | No | Yes | Yes | Yes | Yes | No | Yes | Yes | Yes | Yes | No |

Consolidated Criteria for Reporting Qualitative Studies (COREQ) [21]. Domain 2: study design, the criteria are (9) method orientation, (10) sampling, (11) method of approach, (12) sample size, (13) non participation, (14) data collection, (15) non-participants present, (16) sample description, (17) interview guide, characteristics, (18) repeat interviews, (19) recording, (20) field notes, (22) data saturation, (23) transcripts returned.

| **First author** | **Year** | **Domain 3: analysis and findings** | | | | | | | | | |
| --- | --- | --- | --- | --- | --- | --- | --- | --- | --- | --- | --- |
|  |  | **Data analysis** | | | | | **Reporting** | | | | |
|  |  | **24** | **25** | **26** | **27** | **28** | **29** | **30** | **31** | **32** | **Conclusion** |
| Berkelmans | 2010 | Yes | No | No | Yes | No | Yes | Yes | Yes | No | Include |
| Ekawati | 2017 | Yes | No | No | No | No | Yes | Yes | Yes | No | Include |
| Marcinowicz | 2014 | Yes | No | Yes | No | No | Yes | Yes | Yes | Yes | Include |
| Bjorkman | 2009 | Yes | No | Yes | Yes | No | Yes | Yes | Yes | No | Include |
| Chauhan | 2018 | Yes | Yes | Yes | Yes | No | Yes | Yes | Yes | Yes | Include |
| Pepp | 2014 | Yes | No | Yes | No | No | Yes | Yes | Yes | No | Include |
| Naidoo | 2019 | Yes | No | Yes | Yes | No | Yes | Yes | Yes | Yes | Include |
| Ross | 2015 | Yes | No | Yes | Yes | No | Yes | Yes | Yes | Yes | Include |
| Artuso | 2013 | Yes | Yes | Yes | Yes | No | Yes | Yes | Yes | Yes | Include |

Consolidated Criteria for Reporting Qualitative Studies (COREQ) [21]. Domain 3: analysis and findings, the criteria are (24) number of data coders, (25) coding tree description, (26) derivation of themes, (27) software, (28) participant checking, (29) quotations, (30) consistency data and findings, (31) major themes clarity, (32) minor themes clarity. Articles were included if there were no serious concerns about the quality and approved by all authors.
